# Language skills in Parkinson’s disease: state of the art

**DOI:** 10.1590/1980-5764-DN-2025-0350

**Published:** 2026-02-06

**Authors:** Guilherme Briczinski de Souza, Bárbara Costa Beber

**Affiliations:** 1 Universidade Federal de Ciências da Saúde de Porto Alegre, Programa de Pós-Graduação em Ciências da Reabilitação, Porto Alegre RS, Brazil.; 2 Universidade Federal de Ciências da Saúde de Porto Alegre, Departamento de Fonoaudiologia, Porto Alegre RS, Brazil.

**Keywords:** Parkinson Disease, Neuropsychology, Speech Disorders, Language Disorders, Communication, Cognition, Doença de Parkinson, Neuropsicologia, Distúrbios da Fala, Transtornos de Linguagem, Comunicação, Cognição

## Abstract

This review aims to examine the state of the art regarding changes in language abilities in Parkinson’s disease (PD), with a focus on different linguistic domains. To this end, the language alterations are organized into sections (phonetic and phonology, morphology, syntax, semantic and lexicon, and pragmatics) that correspond to each linguistic domain and are complemented by considerations for future research. Linguistic changes can emerge early in the course of PD, even when verbal abilities are not yet significantly compromised. As the disease progresses, individuals with PD commonly exhibit executive dysfunction, visuospatial difficulties, and memory impairment. Communication deficits extend beyond speech and voice, and are directly impacted by linguistic functions. Given the progressive nature of PD, the study of language provides valuable insights into the impact of neurodegeneration on communication, an area often overshadowed by the predominant focus on motor speech disorders, such as dysarthria.

## RESUMO.

Esta revisão tem como objetivo examinar o estado da arte sobre as alterações nas habilidades linguísticas na Doença de Parkinson (DP), com foco nos diferentes domínios da linguagem. Para isso, as alterações linguísticas estão organizadas em seções (fonética e fonologia, morfologia, sintaxe, semântica e léxico, e pragmática) que correspondem a cada domínio linguístico e são complementadas por considerações para pesquisas futuras. Alterações linguísticas podem surgir precocemente na DP, mesmo quando as habilidades verbais ainda não estão significativamente comprometidas. À medida que a doença progride, pessoas com DP apresentam disfunção executiva, dificuldades visuoespaciais e prejuízo de memória. Os comprometimentos na comunicação vão além da fala e da voz, sendo também impactados pelas funções linguísticas. Dado o caráter progressivo da DP, o estudo da linguagem oferece importantes *insights* sobre os efeitos da neurodegeneração na comunicação, aspecto muitas vezes ofuscado pelo foco nas alterações motoras, como a disartria.

## INTRODUCTION

 Parkinson’s disease (PD) is characterized by involuntary resting tremors, muscle rigidity, and slowness of movement^1^. The neuropathology of PD involves the loss of dopaminergic cells in the substantia nigra, which projects to the striatum, a key component of the basal ganglia^2,3^. As the disease progresses, other brain regions become impaired, eventually affecting the neocortex^3^. Although motor symptoms are predominant in PD patients, cognitive, emotional, and systemic impairments also occur^4,5^. 

 Regarding cognitive symptoms, people with PD (pwPD) experience a range of deficits as the disease progresses, including executive dysfunction, visuospatial difficulties, and memory impairments^6,7^. In some cases, these changes evolve into patterns consistent with dementia, reflecting the heterogeneity of cognitive impairment in PD. This variability may be associated with distinct underlying neuropathological mechanisms across pwPD. Since these cognitive functions are essential for language processing, their decline may contribute to the language deficits observed in PD^8-10^. 

 Approximately 90% of pwPD experience communication difficulties. Standard references often summarize PD leads to speech impairments as reduced speech volume, pitch fluctuation, breathiness, tremor, hoarseness (roughness), inconsistent speech rates, and imprecise articulation^11-13^. However, communication challenges in PD go beyond dysarthria impairments, and are also related to linguistic functions^14-18^. 

 Changes in linguistic abilities can emerge early in the course of the disease, even when global cognition and verbal skills, such as naming, verbal conceptual judgment, and semantic verbal fluency, are still preserved^14-15^. Recent studies highlight that, in PD, linguistic impairments involve multiple domains of language processing, reflected in changes in morphosyntactic processing, lexical retrieval, and pragmatic competence^14-18^. 

 Understanding these deficits is crucial, as they affect both communication and quality of life. Given the progressive nature of PD, studying language impairments provides valuable insights into how neurodegeneration affects communication, an area often overshadowed by research on dysarthria. This review examines the current state of the art on linguistic abilities impairments in idiopathic PD, with a particular focus on different linguistic domains. A glossary of key terms used in this review can be found in Table 1. 

**Table 1:** Glossary of key terms.

Term	Definition
Language	It is a structured system of symbols — such as sounds, gestures, or writing — governed by rules that relate these forms to meaning, enabling communication and the expression of ideas for a specific purpose.
Dysarthria	It is a speech disorder resulting from lesions in the nervous system that affect some of the motor processes involved in speech production: breathing, phonation, resonance, articulation and prosody, compromising the quality and intelligibility of speech.
Phonetics	It is the systematic study of speech sounds, focusing on the sounds themselves by examining how they are produced, how they are perceived, and the physical aspects involved in their production.
Phonology	It is concerned with the systems and patterns that speech sounds follow.
Prosody	It refers to how speech is rhythmically and melodically structured.
Morphology	It refers to the rules of structure and formation of words. It focuses on how words are built.
Syntaxis	It comprises the rules for ordering words in linguistically meaningful ways.
Semantic and Lexicon	Semantics approaches the meaning in language. It examines how words, phrases, and sentences convey meaning, both individually and in combination. Lexicon is the mental repository of words and their meanings in a language.
Anomia	It is a term used to designate a difficulty or inability to name words, especially names of objects, people, places or concepts.
Verbal Fluency	It is a neuropsychological measure that involves asking the individual to produce, within a limited time (usually one minute), as many words as possible based on a predefined criterion, such as a semantic category (e.g., animals) or an initial letter (e.g., words beginning with the letter F).
Pragmatics	It refers to the human capability to communicate in different contexts, with different interlocutors, recognizing and expressing communicative intentions.

### Phonetic and phonology

 Speech is an act of language and one of its forms of expression. It is a fine motor skill, performed with precision and speed, organized into four levels: linguistic-symbolic planning, sensorimotor planning, motor programming, and motor execution^19,20^. Therefore, we can highlight that phonetics is the systematic study of speech sounds considering how they are produced, perceived and what physical aspects are involved in their production, while phonology is the study of the sound systems of languages and how these sounds are organized, standardized and used to convey meanings^21^. 

 In PD, speech production is affected due to a failure in motor programming, which causes dysarthria^12^. This speech disorder compromises speech intelligibility and quality by affecting breathing, airflow, articulation, and laryngeal function^12,13,22^. These impairments result from neuromuscular problems that impair cooperation and precision of speech movements and directly impact the phonetic aspects^12,13^. 

 The phonetic impairments in PD are the result of a reduction in the amplitude and speed of articulatory movements, resulting in imprecise and indistinct phonemes^23,24^. Phonetic impairment affects different phonemic groups unequally, with occlusive, fricative and affricate sounds being particularly affected^25^. This pattern suggests greater difficulty in executing fine articulatory movements, which are essential for the production of phonetically complex sounds. This reflects a direct impact on the phonetic aspects of speech, such as the precision and clarity of articulation^24-27^. 

 The effects of PD on phonetics are also influenced by both internal and external patient factors. For example, individuals often balance phonetic intelligibility with articulatory effort, adjusting their speech depending on the communicative context^28,29^. In the early stages of PD, articulatory movements may be fast, but the overall speech rate tends to be slower due to the increased number of pauses and difficulties in coordinating phonemes^26,30,31^. These coordination difficulties arise from impairments in the neuromuscular execution of speech, specifically involving weakness, slowness, or incoordination of the articulatory muscles (tongue, lips, jaw, and velum) as well as respiratory and laryngeal systems. Such deficits interfere with the ability to produce accurate and consistent movements, often leading to imprecise articulation, changes in speech rate, reduced coarticulation, and decreased overall speech intelligibility^26,30-33^. 

 Although phonology is not widely investigated in PD, some studies indicate subtle impairments in tasks that require conscious manipulation of phonemes or phonological working memory, suggesting specific difficulties in phonological processing^34,35^. One study showed that healthy individuals tend to outperform those with PD in these tasks, and that such differences cannot be attributed to other cognitive domains^34^. Furthermore, several authors suggest that these phonological deficits may be related to dysregulation of frontostriatal circuits^34,35^. 

 The impact of dopaminergic treatment, such as levodopa, on phonetics remains unclear and seems to vary across individuals. Although there is an improvement in articulatory agility in some cases, the benefits do not translate into improvements in acoustic measures, such as vowel space^28,36^. 

 In conclusion, impaired in the phonetic domains in PD is closely linked to speech dysfunctions that are influenced by muscle rigidity, bradykinesia (slowness of movement), and tremor^13,14,18^. These dysfunctions hinder the precise execution and timing of articulatory gestures, reducing the clarity of phoneme production and altering segmental accuracy during spontaneous speech, when planning demands and rapid phoneme transitions are required^13,14,18,24-27,29^. Consequently, individuals with PD often modify their articulatory strategies, adjusting phonetic patterns and phonological processes according to symptom severity and communicative context^37^. 

### Morphology

 Literature on word morphology in PD is scarce. Several studies are dedicated to analyzing verb inflection^38-41^. The literature shows that pwPD have specific difficulties in verb inflection and verb production, involving motor neural networks and procedural memory in linguistic processing^38-40^. 

 In a study about English speakers with PD, the production of the regular past tense was more difficult than the production of irregular past tense. One possible explanation for this finding is that regular forms require the application of morphological rules, such as adding the *-ed* suffix, which may depend on procedural memory, a function associated with the basal ganglia and its connections with the frontal lobe — structures that are compromised in PD^40^. However, another study found that verbal production ability did not differ between PD and control groups as a function of verb regularity or working memory. Given that verb regularity and working memory were not significant factors, the authors proposed that the observed difficulties in verbal production in PD might instead stem from deficits in executive functioning^41^. 

 Additional evidence reported that pwPD that use dopaminergic medication produce more verbs than those who were not taking medication. The study suggests that dopaminergic networks from the basal ganglia to the affected motor areas may play a role in the retrieval of action verbs with specific semantic representations^42^. 

 In conclusion, despite the limited literature, the evidence suggests that pwPD use fewer morphemes per sentence than controls. Furthermore, pwPD seems to produce less verbs per utterance than the control group, while the proportion of nouns was higher. The studies point that the verbal production is affected due to dysfunctions in the functioning of frontostriatal circuits and dopamine availability^38-43^. 

### Syntax

 The literature on syntax production in PD presents divergent results^15,17,44,45^. Some studies have not found significant deficits in syntax production compared to controls, suggesting that syntax production may be less impaired than syntax comprehension in pwPD^17,44^. However, other studies indicate that pwPD tend to produce speech with lower syntactic complexity, especially in the presence of cognitive impairment^15,45^. 

 In narrative tasks, pwPD produced longer but less complex sentences, without incomplete or incorrect sentences^45,46^. Their speech was observed to occur in short segments, organized in a list-like manner, which may reflect a compensation strategy for motor or cognitive deficits^47-49^. Furthermore, pwPD paused more in syntactically complex sentences than healthy individuals, suggesting a difficulty in the grammatical organization of discourse^45-49^. 

 One possible explanation presented by the studies^18,45-49^ for the reduction in syntactic complexity is that motor limitations in speech lead to a decrease in verbal production, making it more difficult to detect differences between PD and other diseases. Some studies^47,50,51^ interpret this reduction as a strategy to convey relevant information in less time, prioritizing the production of nouns and verbs over morphemes, prepositions, and conjunctions, which are essential for the construction of more elaborate sentences. 

 Other studies have shown that pwPD produce fewer subordinate clauses compared to healthy individuals, although the production of simple sentences remains unchanged^15,50^. On the other hand, there is research reporting the opposite: an increase in the use of subordinate conjunctions and dependent clauses, which has been interpreted as a compensatory strategy for cognitive deficits such as working memory difficulties^17,51^. 

 Syntactic comprehension is also altered in PD due to grammatical and syntactic deficits. However, some studies have shown that attention and executive functions influence performance^52-54^. Therefore, pwPD tend to have difficulty understanding sentences whose order of elements is altered, as occurs in passive sentences, where the subject of the action appears after the object^55-57^. The studies explain that pwPD have difficulty separating themselves from the subject-verb-object syntactic model, presenting limitations in executive control mechanisms, in addition to which it may indicate that they resort to random strategies to understand sentences^52-57^. 

 Studies show that pwPD have difficulties identifying the syntactic function of nouns within sentences and processing more complex sentence structures, which demonstrates deficits in understanding different levels of syntactic complexity in PD^58-60^. Research also demonstrates that syntactic comprehension performance is supported by the recruitment of additional specific regions, especially in sentences that involve greater interaction between semantic processing and syntactic structure, evidencing the presence of compensatory mechanisms that maintain linguistic comprehension despite the difficulties imposed by the disease^61-63^. 

### Semantic and lexicon

 Difficulty finding words and anomia are common symptoms in several neurological diseases and brain injuries. These difficulties impact communication, and can lead to choosing the wrong words in speech, problems formulating ideas and frequent pauses during conversation^64-66^. 

 The literature indicates that pwPD perform worse than healthy individuals in naming manipulable objects, while their performance in naming non-manipulable objects remains similar to that of healthy individuals^18,67^. Furthermore, picture naming tasks revealed deficits in action naming, which remained evident even when patients were under the effect of dopaminergic medication^68^. However, the exact nature of the anomia is controversial, with hypotheses as to whether it derives from difficulties in lexical retrieval or from an impairment of semantic memory^69-70^. 

 Another task that has been widely researched is verbal fluency^71-81^. This task requires executive functions and semantic, phonological, and grammatical analyses for the rapid and appropriate selection of words within a limited time^71^. The literature suggests that semantic fluency is more compromised than phonemic fluency in PD^71^, but there are studies that show that phonemic fluency is more compromised than semantic fluency^72,73^. Such deficits in phonemic fluency may be influenced by educational level and different patterns of distribution of the pathology in the brain^71,74^. Furthermore, aspects such as age, time of disease progression, presence of associated cognitive impairment, use of dopaminergic medication, and individual variability in neural plasticity can also impact performance in this skill^70,75-78^. 

 The nature and progression of verbal fluency deficits in PD appear to be related to the stage of the disease. Some research suggests that reduced verbal fluency may be a marker of conversion to early-stage dementia in PD^76-77^. In the early stages, bradykinesia may be the main limiting factor, while, in more advanced stages, cognitive deficits become more evident^76-77^. Furthermore, linguistic, and semantic deficits, especially those related to the production of action verbs and the semantics of actions, appear early, even in the absence of significant cognitive or executive dysfunctions^76-78^. 

 Studies show that patients with PD have greater impairment in action fluency, while their performance in categorical fluency tasks and lexical fluency tasks is similar of control subjects^77,79^. Furthermore, when comparing performance in verbal and phonemic fluency tasks between subjects with and without dopaminergic medication, it is observed that those in the OFF state present deficits in both tasks, while in the ON state performance returns to normal. These findings reinforce the influence of dopamine on lexical processes^70,75^. 

 Semantic fluency is associated with verbal ability and activation of brain areas such as the anterior region of the left middle frontal gyrus and posterior regions of the temporal cortex, especially the left fusiform gyrus^78^. Impaired semantic fluency in PD may be attributed to deficits in semantic memory and cognitive switching ability^78^. Studies suggest that verbal fluency may be a marker of dysfunction in the frontostriatal circuit, as it presents deficits in this task even when other cognitive functions are intact. Furthermore, they point out that efficient word retrieval depends on connections between the frontal cortex and the basal ganglia, and this impairment becomes more evident in contexts that exclude greater executive control, such as conversations with rapid topic changes^78,80,81^. 

### Pragmatics

 Pragmatic skills are essential for effective communication, especially when there is a discrepancy between literal and intended meaning, as in metaphors, ironies, and proverbs^82^. Pragmatic deficits are often associated with right hemisphere lesions and neurological or neuropsychiatric conditions^83^. In PD, pragmatic difficulties affect both language production and comprehension, with significant impact on the ability to sustain communicative interactions^82,84,85^. 

 Pragmatic impairment in PD has been investigated in relation to deficits in attention, short-term memory, and executive functions, and is often associated with these underlying dysfunctions^83^. In addition, pwPD have difficulties in paralinguistic skills crucial for communication, such as recognizing facial expressions and understanding emotions^86^. Specifically, studies indicate that pwPD produce reduced speech, have difficulties in conversational adequacy, and present deficits in prosody and language processing^87^. 

 Studies^86,88^ have shown that pwPD perform worse than healthy individuals in interview, description, and narrative tasks, presenting global pragmatic disorder. These deficits are not explained by articulation problems, but by difficulties in meeting the interlocutor’s needs, which may be related to reduced communicative initiative and insecurity resulting from motor symptoms^86,88^. 

 Pragmatic deficits in PD especially affect the discursive level, compromising both production, which becomes less informative and shows little verbal initiative, and comprehension, with difficulties in inference and processing of narratives and humor^14,88,89^. Patients with PD also present lower efficiency in processing metaphors, possibly due to deficits in working memory and prefrontal cortex functioning^14,88,89^. 

 Although the exact neural basis of pragmatic deficits in PD is not yet fully understood, evidence suggests frontal lobe involvement^86^. However, the relationship between executive functions and pragmatics in PD remains to be further investigated. 

### Considerations for future research

 Understanding the mechanisms underlying linguistic alterations in PD still presents gaps, requiring longitudinal studies that assess the progression of these impairments over time. Investigating how the different phases of the disease affect linguistic domains will allow a more precise approach to intervention. 

 New technological approaches, such as automated speech analysis and the use of vocal biomarkers, can contribute to the early monitoring of linguistic alterations and the personalization of therapies. The development of models based on artificial intelligence to detect altered linguistic patterns also represents a promising field. 

 One of the main aspects to be considered in future research is the influence of individual factors, such as the use of regional dialects, which may impact the results of linguistic assessments. Another relevant factor is the history of speech therapy of participants with PD, as speech therapy rehabilitation may modify linguistic patterns. In addition, most existing studies evaluate patients under the effect of dopaminergic drugs, which may influence the performance of linguistic tasks. Future research should include comparisons between "on" and "off" states to better isolate the impacts of dopamine on language production and comprehension. 

 The increasing use of clinical scales and questionnaires in clinical practice and research raises the need for their validation through objective measures. Much remains to be done to elucidate the mechanisms underlying language deficits in PD to better define them as quantitative markers of the disease. Furthermore, future investigations should determine whether these deficits emerge at sufficiently early stages to be used as diagnostic markers. 

 Another relevant point is the interaction between the motor, cognitive, respiratory, and affective systems in PD. The assessment of speech and language has great potential to provide information about the patient’s clinical status in these domains. However, measurements performed in laboratory and clinical settings may not be sensitive to early changes in the disease or to the variability of deficits in daily communication. In this sense, mobile technologies represent a promising tool for performing objective, frequent and remote assessments of speech and language in patients with PD. 

 Methodological limitations should also be addressed in future studies. The lack of longitudinal data prevents the analysis of individual trajectories of cognitive and linguistic decline. Furthermore, the lack of structural and functional neuroimaging data limits inferences about the neural bases of the observed deficits. The small sample size also represents a challenge, as PD involves different profiles of cognitive functioning. Future studies should explore differences between patients with high and low cognitive reserve, considering their influence on the progression of linguistic deficits. 

 Finally, it is important to consider emotional and psychiatric factors, such as depression, which has a high comorbidity with PD and can influence verbal expression and communicative motivation. The evaluation of these aspects can contribute to a more comprehensive understanding of linguistic deficits in PD and their implications for patients’ quality of life. 

 Therefore, future research should follow a multidisciplinary and integrative approach, exploring different dimensions of linguistic deficits in PD, from neurobiological aspects to clinical and technological implications, for earlier diagnosis and more effective interventions. 

 In conclusion, the study of linguistic ability in PD has revealed a complex and heterogeneous landscape, with communication impairments extending far beyond voice and intelligibility issues. While research in this field is still developing, language difficulties provide critical insights into patients’ cognitive and communicative profiles, with potential implications for diagnosis and intervention. Understanding whether these deficits stem from central language impairments or broader cognitive dysfunctions remains a key challenge. Future studies must continue refining quantitative markers of linguistic decline, aiming for earlier diagnosis and more effective therapeutic strategies tailored to the multifaceted nature of communication disorders in PD. 

## Data Availability

The datasets generated and/or analyzed during the current study are available from the corresponding author upon reasonable request.
